# Alzheimer's Disease: Screening Biomarkers Using Frequency Doubling Technology Visual Field

**DOI:** 10.1155/2013/989583

**Published:** 2013-09-25

**Authors:** Denise A. Valenti

**Affiliations:** Vision Care, 62 Forest Avenue, Quincy, MA 02169, USA

## Abstract

This study was to investigate the feasibility of frequency doubling technology (FDT) visual field testing in Alzheimer's disease (AD) in order to identify early biomarkers of AD in patients already diagnosed with AD and compare the findings to participants not having Alzheimer's disease. This biomarker would be useful in a battery of tests for the early identification of those with AD. It was not the intent to correlate the visual system biomarker with severity of disease, but to determine if the biomarker was present in pass or fail screening criteria. The study showed with very strong significance that the FDT can identify biomarkers of those with AD compared to an age-matched population that does not have AD. FDT is a simple test to take and administer and has been used to screen for eye and retinal diseases such as glaucoma, retinal macular degeneration, and diabetic retinopathy. The results obtained in the FDT readout are analyzed and compared to the age normative database within the system. The FDT ability to screen for AD biomarker in the visual system was significant in those with AD compared to the controls, and the deficits were not related to any ocular pathology.

## 1. Background

The ability to detect light flicker and contrast sensitivity function is impacted early in diseases affecting visual system neural tissues such as in Alzheimer's disease (AD), diabetic retinopathy, macular degeneration, and glaucoma. Light flicker and contrast sensitivity function reductions often occur before other visual processes are impacted in AD such as Snellen acuity. Screening tests that use either light flicker or contrast sensitivity or a combination of the two can be utilized to detect disease before it has resulted in substantial vision loss and before the vision loss impacts quality of life. In the case of AD, the use of such screenings of neurodegenerative processes affecting the visual system can also result in the timely and appropriate referrals in order to make diagnosis of a cognitive impairing disease. The frequency doubling technology visual field test (FDT) combines a stripe pattern light flicker and variable contrast target. The stripes that are variable contrast are vertical sine wave gratings of low spatial frequency (0.25 c/deg) that undergo counter phase flickering at a high temporal frequency of 25 Hz [1]. It is a simple test to take and to administer. The FDT is already used in eye screenings and is considered an important tool for the identification of eye disease and prevention of vision loss [2, 3]. The FDT has several programs internal to the system to perform either simple screens or more complex threshold visual field screening and assessment. For screening of substantial numbers of patients, such as public health fairs, there is a simple detection program that takes one minute per eye. For our purposes, we used the more complex threshold program that takes four minutes per eye. A large scale project used a different type of instrument, a more complex version of FDT, Matrix FDT with smaller five degree targets and with more targets, to screen over 2,000 participants forty years of age or older in the National Health and Nutrition Examination Survey. The Matrix FDT used was found to be reliable in eighty percent of those tested; with large reductions in acuity, glaucoma, and age reported to be the major contributions to unreliable tests [4].

**Figure 1:**
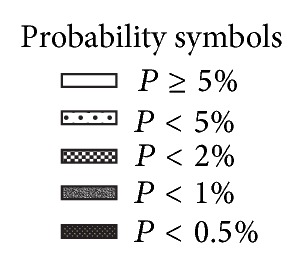
Probability symbols from the frequency doubling technology threshold screening readout.

**Figure 2:**
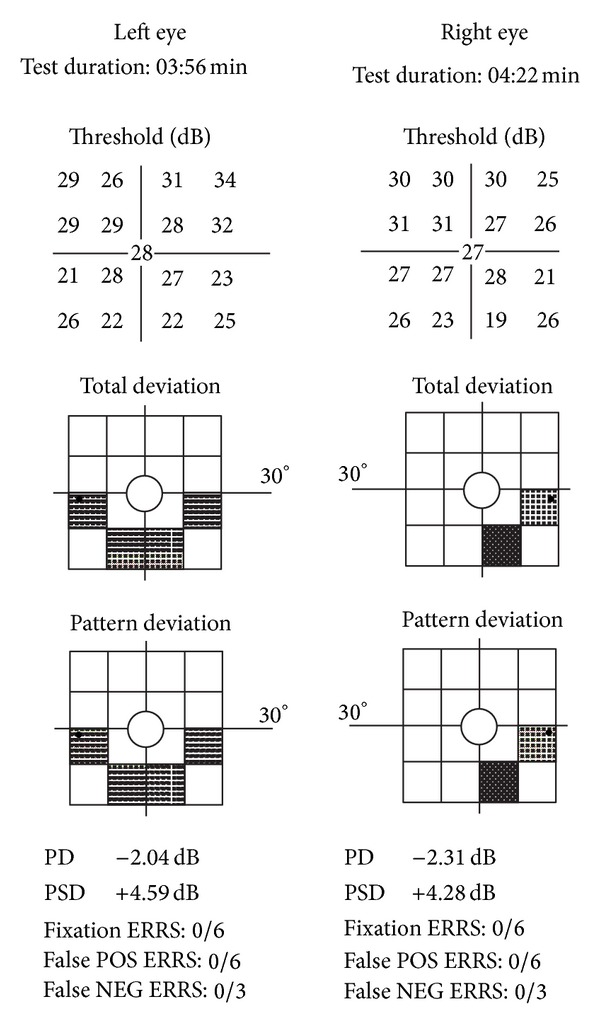
Frequency doubling technology readout of Alzheimer's disease participant. The total deviation was used for the study screening purposes. The bottom of each test shows that for this participant, there were no fixation losses, no false positives, or no false negatives making both tests reliable FDT tests. The left eye had four regions of *P* < 5% and the right eye had one region of *P* < 5%, and one region of *P* < .5%. Both were reliable FDT tests, but failed screenings.

The FDT tests have a built-in age-specific normative database and the readout results are calculated based on this. The test utilizes a low spatial frequency sinusoidal grating that undergoes a high temporal frequency counter phase flicker. When this occurs, the gratings appear as if they are doubled. The contrast is varied during testing using a threshold strategy. The participant views targets that are striped square areas ten degrees in size in either central vision or the same targets extending up to twenty degrees peripheral to the center viewing fixation. There are seventeen targets in all. As with traditional visual field testing, participants are seated and have the forehead positioned in a stabilizing support. The test has the additional benefit of monitoring patient reliability by fixation monitoring tests with computer-generated readouts of fixation losses, false positive errors, and false negative errors. The test is not complex for the patient and is easy to perform even with cognitive impairment or with patients as young as four years [5–7]. While the Matrix FDT used for screening in the National Health and Nutrition Examination Survey had a reliability rate of 80%, the target in that test is smaller being one/half the size; the test has 55 targets, and the spatial frequency of the stripes is smaller. The test is more difficult for participants and it takes longer for participants to perform than the FDT used in this study. The FDT used in the study is easier to administer and take. Studies have shown that patients with mild to moderate AD could perform the FDT test despite their reduced cognitive status [8–10]. A test is considered a successful reliable screening test if there are fewer than a combination of; three fixation losses, false positive errors, or false negative errors, in any combination. A successful reliable screening test is considered a failed screening test if it has readout results of one of the seventeen targets being a *P* < .5% or a *P* < 1% or any two or more of seventeen targets being a *P* < 2% or a *P* < 5% as compared to the age normative database. In an actual screening setting (not research) a failed health screening such as the FDT requires triage and potential referral. If the FDT were to be used in screening to identify biomarkers related to AD and a failure was identified, the triage and needed referrals might involve vision care and/or neurologic consultations.  Figure 1 is the portion of the readout that shows the grey scale and the corresponding *P* values. An example of a failed AD with screening is shown in Figure 2. The participant with the failed FDT screening shown in Figure 2 had a comprehensive dilated eye examination, and there was no ocular pathology within the eye and no pathology identified within the visual system (other than AD) to account for the deficit identified with FDT.

### 1.1. Disease Demographics

Neurodegenerative diseases that are not part of the normal aging process result in tissue damage and loss of brain cells and neuronal connections. AD is the most common age-related degenerative process affecting neural tissue, primarily brain. One of the early sites of AD damage is the brain's visual association area, Brodmann area 19, with loss of cells and neuronal connections [11]. FDT testing identifies cell death and loss of neuronal connections within the visual pathway [12]. The Center for Disease reported that AD was the sixth leading cause of death in 2007 and diabetes was the seventh leading cause of death [13]. Diabetic retinopathy is a leading cause of blindness for those aged 20–74. Macular degeneration is the leading cause of blindness for those over the age of 60 and it is estimated that 11 million people in the United States have macular degeneration [14]. Glaucoma affects approximately 2.8 million Americans [15]. Diabetic retinopathy, macular degeneration, and glaucoma are common diseases associated with aging, and our research population reflected this. The FDT visual field has been used in a screening capacity to detect eye disease, and when a deficit is identified with FDT, a differential diagnosis between eye disease and neurodegenerative processes such as AD is necessary. It is not out of the ordinary for a patient to fail a visual screening due to deficits identified with the FDT and have the eye exam turn out to be considered within normal limits. The FDT as a tool to detect disease originating in the eye early in the visual pathway can also detect disease further along the visual pathway [16] outside the eye such as AD [10, 17–19], Parkinson's disease [20, 21], lesions poster to the chiasm [22], and multiple sclerosis [23, 24]. FDT was found to be better than conventional visual fields in detecting axonal injury due to ischemia along the optic nerve [25]. The FDT has long been considered to be underutilized for applications in the screening and diagnosis of neurologic diseases impacting the visual system [26]. This project demonstrated that the FDT when used as a screening tool most likely can contribute to identifying probable AD.

Contrast sensitivity functions are reduced in neurodegenerative diseases impacting visual pathways. Contrast sensitivity tests can be considered sensitive in detecting pathologies affecting the visual system [27]. Loss of contrast sensitivity function is subtle and not readily detected during standard basic visual assessments and eye exams, but it still impacts quality of life. The FDT visual field has variable contrast and can detect such deficits before other traditional vision tests such as acuity or ophthalmoscopy would identify disease. Deficits in visual contrast sensitivity occur frequently in AD [28–30]. Contrast sensitivity function is also reduced in early glaucoma [31]. Contrast sensitivity function is reduced in patients diagnosed with type two diabetes [32].

Detection of light flicker is diminished in neurodegenerative diseases affecting the visual system. In a study of ten participants with AD compared to participants with no dementia, Mentis and colleagues found significant reductions in the response to light flicker when measured with imaging using positron emission tomography (PET). The stimulus was a grid pattern with increasing temporal frequency. With lower temporal frequencies, there were minimal differences but as the frequencies approached 15 Hz, the reduction in neural response in AD became greater compared to age-matched controls [33]. The FDT proposed for use in this project uses a stimulus of 25 Hz. Another study using the same PET protocol, but investigating the flicker response to light in those with mild dementia in AD, identified a reduced response in participants with mild AD using a high temporal frequency visual stimulation [34]. Using a target of flicker, and motion Kurylo and colleagues found that the deficits in 14 participants with AD compared to 20 age-matched controls approached significance [35]. In their study of critical flicker tests, Curran and colleagues found that those participants with AD had reduced ability to determine flicker in descending mode (when the flicker rate is gradually decreased until one can detect flicker) compared to normal elderly [36]. The authors concluded that a test of light licker, as psychophysical threshold, was free from educational and cultural bias and there are no floor or ceiling effects. They also found that light flicker testing had high test retest reliability and no difficulty administering to dementia patients [37]. A flicker test that included contrast found significant reductions in those with Parkinson's disease compared to age-matched controls [38]. The target used in those studies flickered at 25 Hz and was ten degrees in size and was identical to that being used in the FDT for the studies being reported in this paper. Studies using the FDT instrument have also found deficits in those with Parkinson's disease compared to control participants [17, 21]. Light flicker in the form of critical flicker frequency has been shown to be useful in the detection of neuropathology of the visual system including lesions of the optic nerve [39]. A five-degree flickering target at a rate of 30–40 Hz detected functional deficits in those with glaucoma prior to detection with conventional visual fields in 90% of participants [40].

### 1.2. FDT in Neurodegenerative Disease

FDT has been reported to be useful in AD [41] as its target utilizes low spatial frequencies. Low spatial frequencies are deficit in AD [42, 43] and can be considered a biomarker of disease when identified with FDT. In a study using a Matrix FDT; with smaller targets at a slower flicker rate, Risacher and colleagues found that among the nine participants with mild AD, twenty-seven with mild cognitive impairment and twenty-five age-matched healthy controls, that the AD and mildly cognitive impaired participants had deficits compared to the age matched controls. While this group did control for ocular pathology, they did not exclude unreliable tests. Unreliable tests can include both false positive and false negative tests results, and the work undertaken by Risacher discusses this [44]. Pepin and colleagues also reported the Matrix FDT model to be promising in identifying mild AD, mild cognitive impairment compared to age-matched healthy controls when evaluating the overall visual field deficits [8].

In a study using FDT, there were significant deficits in the inferior portion of the left eye visual field as tested by FDT in six participants with AD compared to age-matched controls [45]. This correlates with reports of superior axonal nerve defects identified by optical coherence tomography imaging which is a structural measure of the neural retinal tissue and optic nerve portion of the visual pathway [46–48]. The representative FDT readout for AD in Figure 2 depicts inferior visual field deficits in both eyes not attributed to any identified eye pathology, and the participant had superior never fiber defects as measured by Optical Coherence Tomography, not attributed to any pathology. The Optical Coherence Tomography images have been previously published [47, 48].

FDT is an established test to screen for glaucoma but is also an excellent visual field screening test for optic neuropathies unrelated to glaucoma as indicated by studies, and the FDT is considered to be underutilized for screening of neurologic disease in general [26]. FDT was found to have a specificity of 100% and sensitivity of 84% when used to evaluate 138 eyes of 103 patients in identifying neuroophthalmological pathology when using pass fail criteria of two or more points being depressed to a *P* less than 1% [49]. In another study using FDT to identify neuropathology that had been confirmed with either MRI, computed tomography, or neuroophthalmological exam, the specificity and sensitivity were comparable to other standard visual testing [49].

## 2. Methodology

The screening data was drawn from two groups of participants tested with the FDT, 23 with AD and 14 control participants. One group was a convenience sample study of adult patients attending clinics at the Boston University Neurology Associates. Anyone attending the clinics at the Boston University Neurology Associates was invited to participate in the visual screening project. The sites for the screening included The Doctor's Office Building Neurology located in Boston, MA, USA and the Boston University Neurology Associates in Weymouth, MA, USA. Family members or other adults accompanying patients were also invited to participate in the screening as control participants. The screening included a brief ocular history, monocular distance visual acuities using a Lighthouse Feinbloom Low Vision Chart (a flip chart with testing from 20/10 to 20/1000 that can be administered at variable distances), and FDT visual field utilizing the 20–1 threshold strategy. All AD participants had ophthalmoscopy of the anterior segment to rule out cataract and to confirm pathology as self-reported by participants or the caregivers. Trace cataracts not impinging on visual acuity do not impact the FDT testing; however, more significant cataracts or media opacity that reduce acuities will impact the testing [50]. An aspect of ophthalmoscopy is the assessment of the anterior structures including the cornea and pupil to screen for abnormalities or opacities, and no participant had anterior segment abnormalities that would impact visual functions such as acuity and contrast or peripheral vision. The participants reporting glaucoma in the screening group (not having dilated eye exams) had miotic pupils making a clear view of the optic nerve difficult, and without a dilated exam the glaucoma diagnosis could not be definitively confirmed in those reporting glaucoma. Self-reported macular degeneration was confirmed by ophthalmoscopy despite miotic pupils. The healthy control participants did not have ophthalmoscopy. The second group of participants was a group of AD and controls that had, as part of a separate protocol, cognitive assessment using minimental status exam and a comprehensive eye examination that included refraction for glasses, ocular motility evaluation, applanation tonometry, slit lamp anterior segment examination, dilated fundus examination, peripheral visual field assessment, and in some cases imaging with Optical Coherence Tomography. A successfully performed screening was considered a fail if any of the seventeen high flicker frequency contrast targets was recorded (using the instrument normative screening printout) as having a probability of *P* < .5% or *P* < 1% or any two or more targets having a *P* < 2% or *P* < 5% as identified in the FDT printout (results based on the technology internal normative database). An FDT test with three or more reliability parameters, fixation loss, false positive or false negative, was considered unreliable and not used in data analysis.

A visual acuity of worse than 20/60 was also considered a failure for that eye. The 20/60 acuity was for this project purpose only as it is a screening protocol, and that is what is considered a fail in large scale public health vision screenings. For the population within the group not having a full eye exam, we did not rule out that the reductions in acuity were due to changes in refraction requiring an update in the glasses. In general to determine if an AD-related pathology is contributing to functional deficits in the visual system (rather than eye disease) requires a careful refraction and a more rigid cutoff of 20/25 in each eye separately and 20/25 binocularly in order to assure that undetected eye pathology or uncorrected refractive error is not contributing to the reductions in acuity. While binocular acuities, with both eyes tested open only at distance, are frequently used for vision research related to AD, they cannot be considered acceptable. If a binocular acuity is the single measure of acuity, acuity measurement can fail to identify an eye that is substantially impaired due to eye pathology because the eye without pathology has not been occluded and is functioning. Undetected eye pathology or uncorrected vision can result in research findings that are inappropriately attributed to AD in studies involving neuropsychological testing. Further in an assessment involving any near testing (this study did not include near testing), refractions for optimum eyeglass use would be necessary to insure that the most appropriate optical corrections both near and far are being used. When the glasses used for near testing are out of date, not adequate in strength or otherwise inaccurate, this results in inaccurate near performance during neuropsychological testing, and this was demonstrated by Bertone and colleagues [51]. The FDT is in general not impacted by uncorrected vision, and the results do not vary depending on refractive errors less than six diopters. The participants used their habitual glasses during the FDT testing. Because the screening protocol does not involve any near tasks and the participants did not require optimum correction for accuracy, an assessment for glasses was not done for the convenience group, and refraction was not part of this screening protocol.

### 2.1. Participant Descriptions

The age range of the twenty three AD participants was 68 to 88 years with the average being 80, and the average for successful tests was 81. There were fourteen females and five males and the gender of four was not recorded. All but one of the AD participants were Caucasians. One AD participant was Native American. The age range of the fourteen control participants was 60 to 81 with the average age being 71. There were nine females, and four males, and the gender of one was not recorded. Two of the participants were African American and twelve were Caucasian. The groups receiving the comprehensive eye examinations were part of a separate protocol involving evaluation with FDT and were recruited from a clinical neurology population, Alzheimer's disease registries, support groups, and enrollees of adult day care. The controls and all but the AD participants enrolled in day care received a comprehensive eye examination on campus by a single provider on the same day as the FDT testing. The day care participants received a comprehensive exam through contract with nursing home eye care providers but did not have peripheral visual field testing as part of the comprehensive evaluation. During a separate session and prior to testing with FDT and prior to having a peripheral visual field evaluation these participants had repeat acuities, ophthalmoscopy, and slit lamp anterior segment evaluations (these and the visual field were done using customized portable nursing home examination equipment). The study followed the tenets of the Declaration of Helsinki, and the protocol was approved by the Boston University Institutional Review Board. All participants and, when appropriate, caregivers were provided with written informed consent before participation in the screening study. All participants signed HIPPA approved record request forms and record release forms in order to triage identified ocular pathology as needed.

### 2.2. Apparatus

FDT presents back-lit flashed images viewed on a fixed, flat, shielded screen in front of a stationary participant. The instrument has readouts that are based on normative data, and the internal data is also age normed taking the reported age into account when the internal calculations are made. The participant views targets that are small striped square-shaped areas in either central vision or targets extending up to twenty degrees peripheral to fixation. As with traditional visual field testing, participants are seated and have the forehead positioned in a stabilizing support. Participants are tested monocularly with their habitual eyeglass correction. It is not necessary to patch or otherwise cover the untested eye as the instrument is designed for passive occlusion. This is of benefit to an aged cognitively impaired population who may present with agitation. The participants fixate a target directly in front of them and respond by pushing a button each time they see an image flashed anywhere in their visual field. The instrument records and retests areas based on the participant responses. Care was taken to assure that there was adequate hand coordination to perform the task. The response button was in the better functioning hand as reported by the participant. The instrument always tests the right eye first followed by the left eye. The technology tests for false positive and false negative rates as well as fixation losses. The test has the additional benefit of monitoring patient reliability by fixation monitoring with computer-generated readouts of the fixation losses, false positive errors, and false negative errors. The test takes four minutes per eye. The test is portable, and the testing in the neurology clinic was done in an ancillary conference area. The FDT instrument was set up on a counter height fold stool, and participants were seated in a clinicians stool with a back. The FDT was placed on a desk in the eye examination area during testing of those participants receiving comprehensive evaluations. The clinician's examination chair with a back was used for testing.

In a clinical setting, the standard would be to repeat the unreliable test as there can be attention and learning aspect to the visual field testing, and the second test may yield a more reliable test. When using the FDT for screening in large groups, the test is often not repeated. The FDT was administered only once to all but two AD participants. The FDT was repeated a second time during the same session in the AD participant diagnosed with probable glaucoma and the right eye test of a different AD participant was repeated due to technical problems with the response button.

## 3. Results 

There was a screening failure rate of 87% with the FDT visual field in the participants with AD while only 8% of the control group failed the screening. The AD group had a rate of reported ocular pathology of 37%, and the control group had ocular related pathology at 21%. The breakdown of useable tests appears in Table 2. Utilizing a chi-square test of independence, the overall failure rate when screened with FDT and excluding the unreliable tests for those with AD was significantly greater in comparison to the control group, *X* ^2^(1) = 25.63, *P* < .0000004. The failure rates, when controlling for pathology by exclusion in addition to excluding unreliable tests, were also significant for those with AD compared to the control group, *X* ^2^(1) = 22.56, *P* < .000002. The overall rate of pathology, although higher for the AD group, was not significantly higher *X* ^2^(1) = 0.72, *P* = .40. While the FDT has internal age normative database from which results are generated, to assure this was not a factor the data were analyzed using only those AD and control participants 65 and older and 84 years old or younger. The AD group controlled for reliability, pathology, and age still had significantly higher rates of FDT screening failures compared to the control group, *X* ^2^(1) = 14.85,  *P* = .0001.

The screening results from each eye of participants with a diagnosis of AD were obtained with FDT. There were 23 participants with AD. While 46 eyes were available, five eyes were not tested. Both eyes of one participant were untestable due to severe glaucoma and an inability to fixate the FDT target both eyes of another participant were untestable due to age related macular degeneration and one participant had significant vision loss in only one eye secondary to stroke. The inability to test using FDT had more to do with an eye pathology and inability to perceive the targets than an age-related dementia. Substantially reduced visual acuity due to eye pathology often results in unreliable FDT tests [2]. This can occur with glaucoma due to large deficits in visual field, even when reasonable acuity is identified. Visual field loss in eye disease is a factor contributing to unreliable tests with FDT and poor performance. The control participants were drawn from adults not scheduled as a patient in the neurology clinic, but accompanying a patient or from the control participants that were recruited for participation in other studies at the university. There were 14 control participants, and 28 eyes were tested, and only two of the 28 eyes tested were unreliable. Of 41 eyes of those with AD tested with the FDT, eleven were unreliable. In this study, some of the unreliable tests may have been secondary to dementia, and unreliable tests were defined as a combination of three or more of fixation loss, false positive, and false negative responses. Of the 11 unreliable FDT tests that were successfully done, only one had association with reported ocular pathology, and this was stroke. After the exclusion of unreliable tests, there were final 30 successful FDT screening tests in the AD participant group. Criteria for a deficit in visual function with FDT were deficits in two targets and any test showing reductions based on these criteria was considered a successful FDT but a failed screening test. A reduced acuity was also considered a screening failure, but failed acuities were to be expected in a population known to have ocular pathology. The population evaluated with comprehensive eye examination was prescreened for eye disease over the telephone. There were two cases of undiagnosed glaucoma in the prescreened group, one in the AD and one in the control group. The convenience population was not prescreened for pathology. Failed acuity is not a biomarker of AD. Traditionally, a failed visual screening with FDT indicates pathology in the visual system, but a failure can be in the eye or due to degenerative disease that affects the visual system beyond the eye in the neuroprocessing visual pathways such as AD. Twenty-six of the 30 successful reliable FDT tests obtained from AD participants were considered to be failed FDT screening tests. Seven failed FDT were attributed to a reported glaucoma (some confirmed by ophthalmoscopy), and two failures could be accounted for by dense cataracts (confirmed by ophthalmoscopy). Seventeen FDT failures were not associated with any reported ocular pathology of the eye (majority confirmed by ophthalmoscopy). Of failures due to visual acuity, one was related to stroke, two secondary to glaucoma, and two from age-related macular degeneration. Two of the failed visual acuity had no association with reported pathology or any pathology detected with screening ophthalmoscopy. In an actual setting screening for eye pathology and neurodegenerative disease, a failed acuity would be considered a sign of eye disease, not AD, and the primary referral would be for a comprehensive eye exam. Both of the failed acuity in the AD group eyes had also failed the FDT screening. The results of these screening failures were provided to the participants with the advice to report the results immediately to their eye care provider or primary care physician. Of 28 eyes screened with FDT in the control participant group, there were only two unreliable tests and 4 failed FDT. The one failed FDT was in a participant reporting long standing diabetes. An additional participant also with diabetes had a failure due to acuity. The characteristics of the control participants appear in Table 1, and the characteristics of the control participants are in Table 2. Results are summarized in Table 3.

The average age of the control group was nine years younger than AD group. The lower average age of the control group may be reflective of the caregiver role, younger and healthier. However, the age difference is not likely to fully account for the differences in failure rate with FDT, and using data controlled for age, the differences between AD failures compared to controls remain significant. There was a failure rate of 87% in the participants with AD while only 8% of the control group failed the screening. The AD group had a rate of reported ocular pathology of 37%, and the control group had ocular related pathology at 21%. The group participating in this screening study had access to health care, and the majority reported to have comprehensive eye exams in the previous year. After adjusting for unreliable tests and pathology, the failure rate remains high with the AD participant group having a failure rate of 85% compared to the control group failure rate of 1%. When this is corrected for age including only participants between ages 65 and 85, AD participant failure rate remains similarly high at 85% for the AD group and 1% for the age-matched control group.

## 4. Discussion

Health screenings are important in groups that have limited resources or difficulty accessing health care. This is true for a population with cognitive impairments. In a different study investigating the use of FDT as a screening tool for eye disease (not AD), it was demonstrated there were higher rates of previously undetected visual dysfunction or disease in testing venues specializing in care of the elderly such as senior citizen centers [3]. Such failures may be an indication of AD in the absence of eye pathology after eye examination. The significance of failures with FDT in those participants with AD compared to control participants not having AD demonstrated in this project that FDT identifies a biomarker of AD with the technology. Given the ease and efficiency that screening with FDT presents, this instrument may prove to be an effective means of identifying age-related neurodegenerative processes affecting the visual system, specifically AD. AD impacts the visual system, and the biomarker identified with FDT can be part of the differential diagnosis when there are failures during visual screenings utilizing FDT. Screening with FDT not only can be an integral part of the early identification of age-related eye disease, but also neurodegenerative disease impacting the visual system such as AD.

## Figures and Tables

**Table 1:** Characteristics of control participants.

Age	Gender	Race	OD VA	OS VA	MMSE	FDT OD	FDT OS	Reliability	Eye diagnosis
Participants receiving eye screening									
60	Female	Cau	10/10	10/10		Pass	Pass	0/0	None
63	Female	Cau	10/10	10/10		Pass	Pass	fx2/fx2fn1	CA
63	Male	Cau	10/40	10/20		Pass	Pass	0/fx1	Card/DM
66	Female	Cau	7/10	7/10		Pass	Pass	0/0	Laser
66	Female	Cau	7/10	7/10		Pass	Pass	0/0	None
69	Female	AfrAm	7/10	7/10		Pass	Pass	0/0	None
72	Female	AfrAm	7/10	7/10		Pass	Pass	0/0	None
73		Cau	7/10	7/10		Pass	Pass	0/0	None
78	Male	Cau	10/20	10/20		Pass	Fail	0/fx1	DM

Participants receiving comprehensive dilated eye examination and refraction									
71	Male	Cau	Pass	Pass	30	Pass	Fail	0/0	Eye exam WNL
75	Male	Cau	Pass	Pass	30	Pass	Pass	0/0	Eye exam WNL
78	Female	Cau	Pass	Pass	30	Pass	Pass	0/fx2fp1	OD WNL OS ptosis
81	Female	Cau	Pass	Pass	30	Fail	Pass	0/0	OD ptosis OS ptosis
81	Female	Cau	Pass	Pass	30	Fail	Fail	0/0	Glaucoma

**Table 2:** Characteristics of Alzheimer's disease participants.

Age	Gender	Race	OD VA	OS VA	MMSE	FDT OD	FDT OS	Reliability	Eye diagnosis
Participants receiving eye screening and anterior segment screening									
68	Female	Cau				Fail	Fail	0/fn1	None
75	Male	Cau	10/10	10/10		Pass	Fail	0/0	Glaucoma
76	Male	Cau	*7/80 *	7/20		N/D	Fail	—/fx5	Stroke
77		Cau	*7/30 *	7/10		Fail	Fail	0/v2	none
79		Cau	7/10	7/10		Fail	Fail	fn3/fn2	none
80	Female	Cau	10/30	10/30		Fail	Fail	fn1/0	Cataract
81	Female	Cau	7/25	7/25		Fail	Fail	fp1fn2/fx2fp1fn1	None
81	Female	Cau	*7/120 *	*7/120 *				Unable	Macular Degen
81	Female	Cau	10/10	10/10		Fail	Fail	0/fx1	Glaucoma
82		Cau	10/10	10/10		Pass	Pass	0/0	None
83	Female	NatAm	7/20	7/20		Fail	Fail	0/fx1fp2	None
83	Male	Cau	10/20	10/20		Fail	Fail	Fn1/fn1	Glaucoma
84		Cau	*10/40 *	10/30		Fail	Fail	0/fp1 agitated	None
84	Male	Cau	*10/40 *	*10/50 *				Unable	Glaucoma
87	Female	Cau				Fail	Fail	fx2fp1fn2/fp2fn3	Cardiac
88	Female	Cau	10/10	10/20		Fail	Fail	0/0	None

Participants receiving comprehensive dilated eye examination and refraction									
69	Female	Cau	Pass	Pass	15	Fail	Fail	3fn/3fn	Eye exam WNL
70	Female	Cau	Pass	Pass	23	Fail	Fail	0/0	Glaucoma*
72	Female	Cau	Pass	Pass	24	Fail	Fail	0/0	Eye exam WNL*
72	Male	Cau	Pass	Pass	26	Fail	Pass	2fx2fp/5fx5fp	Eye exam WNL*
85	Female	Cau	Pass	Pass	15	Fail	Fail	0/0	Eye exam WNL
85	Female	Cau	Pass	Pass	17	Fail	Fail	0/0	Eye exam WNL
87	Female	Cau	Pass	Pass	20	Pass	Fail	0/0	Eye exam WNL

Key: Cau: Caucasian, NatAm: Native American, AfrAm: African American, fp: False Positive, fn: false negative, fx: fixation loss, glau: glaucoma, AMD: age-related macular degeneration, CA: cancer, card: cardiac, DM: diabetes, and N/D: not done; *participant had comprehensive eye examination that included optical coherence tomography imaging.

**Table 3:** 

Category of analysis	Probability values (two tail)	Chi squared
Failed FDT between groups, nothing excluded	*P* = 1.39941*E* − 08 ***P* < *0.00000001***	32.20
Failed FDT between groups, exclude unreliable	*P* = 4.16763*E* − 07 ***P* < *0.0000004***	25.63
Failed FDT, exclude unreliable and pathology	*P* = 2.04065*E* − 06 ***P* < *0.000002***	22.56
Failed eyes OU or monocular between groups	***P* = *0.01***	5.83
comparison of reliability between groups	***P* = *0.03***	4.62
Comparison of pathology between groups	*P* = 0.40	0.72
Failed FDT, control unreliable/pathology/age	***P* = *0.0001***	14.85
